# Multi-functional adaptor SKAP1: regulator of integrin activation, the stop-signal, and the proliferation of T cells

**DOI:** 10.3389/fimmu.2023.1192838

**Published:** 2023-05-31

**Authors:** Chen Liu, Monika Raab, Yirui Gui, Christopher E. Rudd

**Affiliations:** ^1^ Faculté de Medicine, Université de Montréal, Montréal, QC, Canada; ^2^ Département de Microbiologie, Infectiologie et Immunologie, Université de Montréal, Montréal, QC, Canada; ^3^ Division of Immunology-Oncology, Centre de Recherche de l’Hôpital Maisonneuve-Rosemont, Montréal, QC, Canada; ^4^ Department of Obstetrics and Gynaecology, School of Medicine, J.W. Goethe-University, Frankfurt, Germany; ^5^ Division of Experimental Medicine, McGill University, Montreal, QC, Canada

**Keywords:** T-cells, signalling, adaptor protein, integrin activation, SKAP1

## Abstract

T-cell activation is a complex process involving a network of kinases and downstream molecular scaffolds or adaptors that integrate surface signals with effector functions. One key immune-specific adaptor is Src kinase-associated phosphoprotein 1 (SKAP1), which is also known as src kinase-associated protein of 55 kDa (SKAP55). This mini-review explains how SKAP1 plays multiple roles in regulating integrin activation, the “stop-signal”, and the optimization of the cell cycling of proliferating T cells through interactions with various mediators, including the Polo-like kinase 1 (PLK1). Ongoing research on SKAP1 and its binding partners will likely provide important insights into the regulation of immune function and have implications for the development of new treatments for disease states such as cancer and autoimmunity.

## Introduction

The activation of T cells involves the processing and presentation of peptide antigen bound to class I and II major histocompatibility complex (MHC) antigens on the surface of antigen-presenting cells (APCs) such as dendritic cells (DCs) (1, 2). The initial contact between a DC and a T cell involves random encounters, or responses to chemokines that can partially activate integrins. Integrins are transmembrane receptors that bind key surface ligands such as proteins needed for adhesion between cells or to extracellular matrix proteins. Of the 12 integrins that are expressed on lymphocytes, αLβ2 [leukocyte function-associated antigen-1 (LFA-1), also termed CD11a (αL chain of LFA-1)–CD18 (β2 chain of LFA-1)] binds to the ligands intracellular adhesion molecules 1, 2, and 3 (ICAMs-1, 2 and 3) (3). Adhesion *via* integrins is needed for the migration of T and B cells to different tissues and to sites of inflammation, for movement in lymph nodes and germinal centers and for the conjugation of T cells with APCs.

Importantly, the initial contact of a T cell with an APC ligates the T-cell receptor (TCR) complex that then induces “inside-out” signals for high-avidity adhesion (4). This is accompanied by more stable conjugation and the formation of an interface between the T cells and APCs, termed the “immunological synapse” (IS) (5). The process involves the formation of initial signaling micro-clusters that coalesce to form the supramolecular activation cluster (SMAC) (6, 7). Chemokines can enhance this process and increase the longevity of the adhesion between integrins and ligand (8). Atomic force microscopy has shown that conjugation forces develop over time and are highest when synapse formation is maximal (9).

When the TCR binds to peptide-loaded MHC molecules on APCs, it triggers the transcription of numerous genes related to T-cell development, differentiation, and effector functions. The specific genes transcribed may vary depending on the context such as the type of T cell, the strength and duration of the TCR signal, and the presence of co-stimulatory signals. Some of the most significant genes that are transcribed include IL-2, IFN-γ, TNF-α, CD69, and nuclear factor of activated T cells (NFAT). The earliest events induced by TCR ligation involve the induction of a tyrosine phosphorylation cascade in T cells (10, 11). Tyrosine phosphorylation is a relatively rare event accounting for less than 1% of total phosphorylation in cells (12). Nevertheless, it is crucial to virally induced phosphorylation events and activation by certain growth factor receptors (13). We first documented the binding of src kinase p56^lck^ to human CD4 and CD8, which initiates a phosphorylation cascade and leads to the phosphorylation of the antigen–receptor complex (10, 11, 14). We proposed that the CD4 and CD8 co-receptors would bring p56^lck^ into proximity of the TCR complex due to binding to non-polymorphic sequences in MHC antigens (15, 16). The presence of these activation complexes was also reported in murine T cells (17) while others have underscored a role for free p56^lck^ in the initiation process (18).

The CD4/CD8-p56^lck^ complexes and p56^lck^ alone can then phosphorylate immunoreceptor tyrosine-based activation motifs (ITAMs) in the CD3 and T-cell receptor ζ chains for the recruitment of a second protein kinase ZAP-70 (ζ-chain associated protein kinase-70) kinase (19). Both p56^lck^ and ZAP-70 then phosphorylate the so-called “adaptor” proteins or molecular scaffolds that form complexes that integrate signals from the cell surface to the nucleus (10, 20). p56^lck^ phosphorylates a broader array of substrates than ZAP-70, many of which overlap with the substrates of other src-related kinases (11, 21). The major identified targets of ZAP-70 are immune-cell adaptors termed LAT (linker for activation of T cells) and SLP-76 (Src homology 2 domain-containing leukocyte protein of 76 kDa) (22, 23). ZAP-70 phosphorylates LAT, which recruits GRB2-related adaptor GADS, which in turn binds with high stoichiometry to SLP-76 (22). The LAT complex also recruits the kinase ITK (interleukin-2-inducible T-cell kinase), leading to the phosphorylation of the phospholipase Cγ1 and the mobilization of intracellular calcium (24). Recently, we showed that another integrin-linked kinase, FAK1 (Focal Adhesion Kinase 1), and PYK2 (proline-rich tyrosine kinase-2) could phosphorylate LAT on a specific Y-171 residue for GRB2 binding (25). This is a new model for LFA-1 in which the integrin can mediate both adhesion and de-adhesion events dependent on receptor cross-linking.

In addition to the activation of gene transcription, the TCR must activate integrins such as LFA-1 on the surface of T cells to generate stable conjugation with an APC (26). Integrins are inactive on resting cells but, in the “jackknife model”, unfold to form intermediate- and high-affinity binding (27). LFA-1 has been studied extensively where the αL subunit consists of a binding pocket containing an I-domain, a β-propeller, and an extracellular and cytoplasmic tail. The β2 subunit is composed of an I-like domain that interacts with the β-propeller, and four integrin-epidermal growth factor-like (I-EGF) domains, which acts as a “leg” of the subunit (28). LFA-1 occupancy with surface ICAM-1 is needed for TCR conversion to an open headpiece high-affinity state (29). Interestingly, this appears not to be the case for chemokine-triggered LFA-1. During antigen presentation, LFA-1 is rearranged in a pSMAC ring that surrounds the TCR complex and other receptors in the central IS while binding to the ligand intercellular adhesion molecules (ICAMs) (30).

## SKAP1 and T-cell adhesion

**Figure 1:**
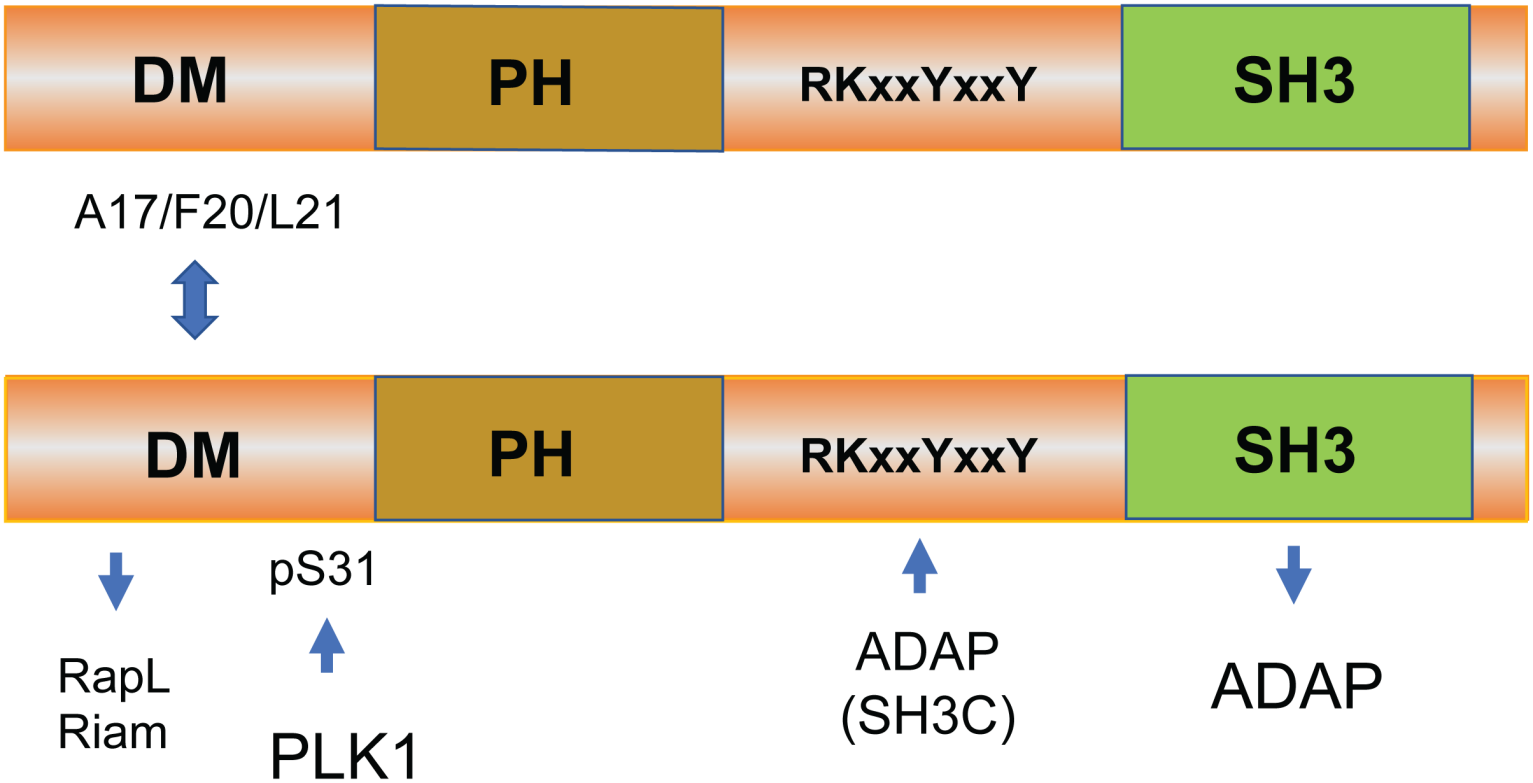
The structure and binding sites of SKAP1. Human SKAP1 possesses an N-terminal dimerization (DM) domain, a species-specific disordered region, a pleckstrin homology (PH) domain (N107–K210), and a C-terminal SH3 domain (D294–E355) (33, 39). SKAP1 binds to ADAP *via* its SH3 domain and, to a lesser extent, *via* a SKAP1 RKxxYxxY motif that binds to an SH3C domain (41, 42). SKAP1 can form homodimers or heterodimers with related SKAP2 (SKAP-related R or SKAP-Hom) in cells mediated by residues A17 to L21 in the SKAP1 N-terminal region (43). The function of dimer formation is not known.

Although many upstream signals are needed to initiate the process (31), the downstream effector proteins in “inside-out” signaling for LFA-1 activation are only partly known. One advance in this area came with the discovery of immune-cell adaptors ADAP [previously known as Fyn T-binding protein (FYB)] and SKAP1. ADAP was cloned independently by the Koretzky and Rudd labs where it binds to the SH2 domain of the upstream adaptor SLP-76 (32–36). ADAP is also a preferred substrate of the src kinase p59^fyn^ (32, 37). SKAP1 was cloned independently by the Schraven and Rudd labs, the latter using ADAP as bait in a two-hybrid screen (33, 38). Human SKAP1 possesses an N-terminal dimerization (DM) domain, a species-specific disordered region, a pleckstrin homology (PH) domain (N107–K210), and a C-terminal SH3 domain (D294–E355) (33, 39). It is an intracellular immune adaptor protein expressed in thymocytes, T cells, and NK cells (40) (**Figure 1**). SKAP1 binds to ADAP *via* its SH3 domain and, to a lesser extent, *via* a SKAP1 RKxxYxxY motif binding to an ADAP SH3C domain (41, 42).

Early transfection and knock-down studies showed that SKAP1 promotes dwell times between T cells and dendritic cells (DCs) (26, 44–49). We showed that SKAP1, but not related SKAP2, regulated TCR-induced lymphocyte-associated antigen-1 (LFA-1) clustering and T cell–APC conjugation (48). No effect on TCR-CD3 clustering was seen (26, 48). SKAP1 enhances adhesion to both fibronectin and intercellular adhesion molecule-1 (ICAM-1), can colocalize with actin at the T cell–APC synapse, and can promote the clustering of LFA-1. The enhanced conjugation was comparable to that seen *via* adhesion and degranulation-promoting adaptor protein (ADAP), a binding partner of SKAP1, and is abrogated by the deletion of the SKAP1 SH3 domain. Conjugate formation is also accompanied by the translocation of SKAP1 to membrane rafts (46, 47). The loss of SKAP1 in *skap1-/-* mice had no obvious effect on thymic development, or the numbers of peripheral T, B, and natural killer (NK) cells. Instead, the *skap1-/-* T cells had impaired binding to ICAM1, more transient conjugation times, and a reduced localization of TCR-CD3 micro-clusters at the IS (49).

**Figure 2:**
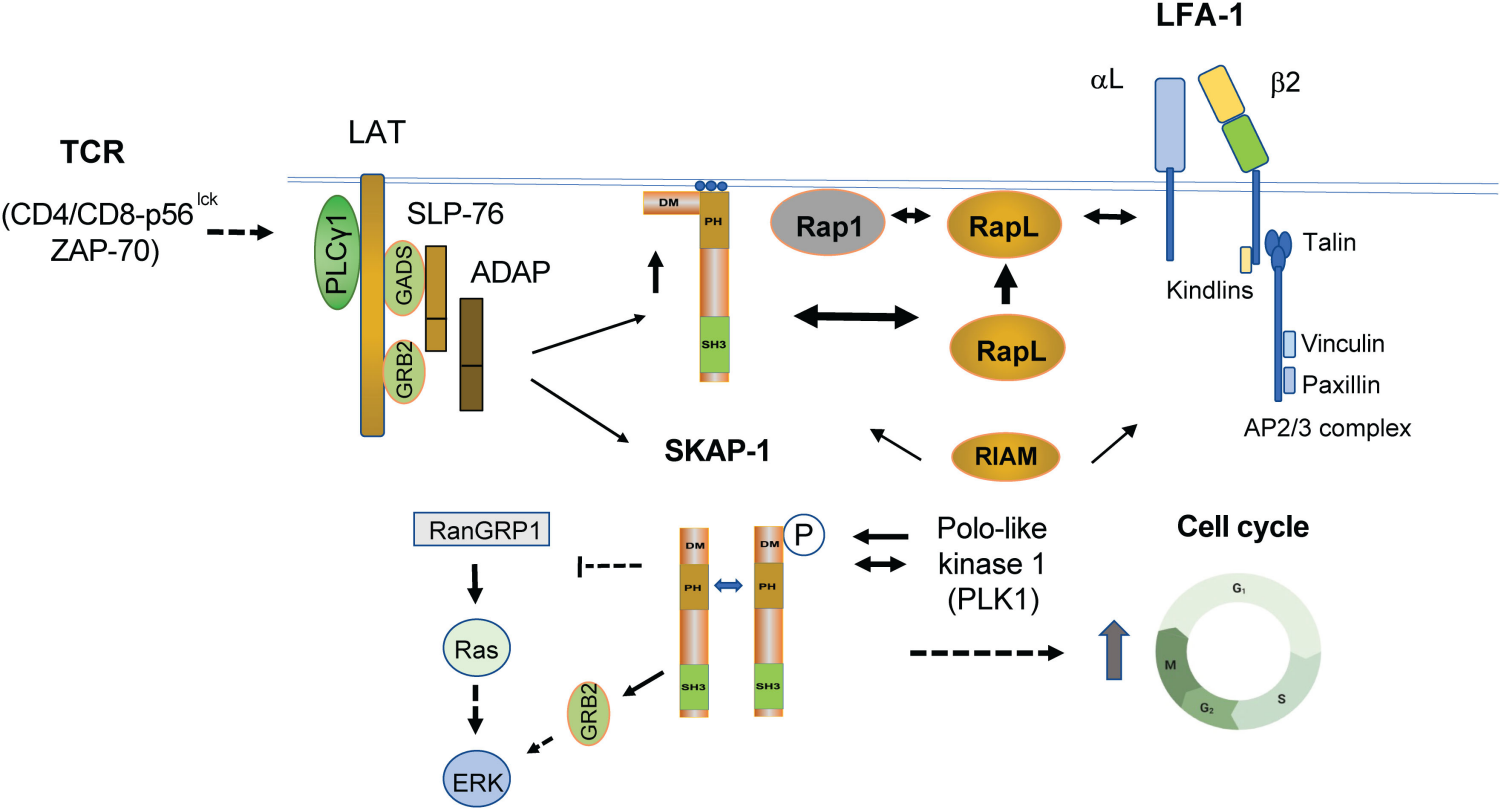
TCR induced pathways linking SKAP1 to the regulation of integrin-mediated adhesion and intracellular proliferation pathways. TCR ligation leads to the formation of the LAT signalosome (LAT and associated SLP-76, GADs, ADAP, and SKAP1). TCR (and CD28) also induces the presence of D-3 lipids, which bind and recruit SKAP1 *via* its PH (pleckstrin homology) domain to the plasma membrane (PM). By binding to RapL in the cytoplasm, SKAP1 acts as a chaperone or shuttle protein to transport RapL to the PM to interact with the GTP-binding protein Rap1 (see upper heavy arrow between SKAP1 and RapL where RapL is moved to the cell surface upward heavy arrow). The RapL–Rap1 complex depends on the presence of SKAP1 as shown by the observation that complex formation fails to form in *skap1-/-* primary T cells in response to TCR ligation. The complex at the cell surface with associated SKAP1 then binds to the αL chain of LFA-1. Concurrently, SKAP1 also binds to RIAM *via* the DM domain. By contrast to the αL chain, the β2 chain binds to Talin and various Kindlins in a complex that includes direct Talin binding to RIAM, Paxillin, and Vinculin (see upper right image of LFA-1). Complex formation promotes increased affinity as well as the clustering of LFA-1 for increased binding avidity in binding to ICAMs. SKAP1 also forms a dimer *via* its DM domain where it regulates the movement of SLP-76 micro-clusters. Whether dimerization controls the binding of RapL and RIAM is not known. In addition to mediating integrin activation, SKAP1 is phosphorylated by and binds to Polo-like kinase (PLK1) for the optimal cycling of T cells (see lower image of SKAP1 dimer). PLK1 binds to the N-terminal residue serine 31 (S31) of SKAP1 and the interaction is needed for optimal PLK1 kinase activity and cell cycling. The C-terminus of SKAP1 also binds to RasGRP1 and can negatively regulate the p21^ras^-ERK pathway or in binding to GRB-2 may promote ERK activation. Whether this occurs alone or in conjunction with GRB-2 binding to LAT remains to be resolved. Solid arrows indicate direct interaction, whereas dotted arrows reflect indirect and unestablished interactions.

Mechanistically, we and others have shown that the adaptor can regulate LFA-1 activation *via* at least two non-exclusive pathways. One pathway involves SKAP1 binding to the SARAH domain of RapL (50–52), an immune cell isoform of the RASSF5 (Ras association domain family 5) family (53). The Kinashi lab has already done seminal work on the importance of Rap1–RapL binding in integrin activation (54, 55). Remarkably, we then found that RapL failed to form complexes with Rap1 in *skap1-/-* T cells and that the SKAP1 PH domain plays a pivotal role in the pathway (44, 50). TCR and CD28 ligation can induce D-3 lipids, phospholipids with a phosphatidylinositol (PI) head group that is phosphorylated on the inositol ring at the 3-position. These lipids bind to the SKAP1 PH domain, thereby promoting its translocation to the inner face of the plasma membrane (56, 57). In this manner, the PH domain allowed SKAP1 to act as a kind of “shuttle” to facilitate the translocation of RapL to the inner face of the plasma membrane where it can interact with the GTP-binding protein Rap1, a potent stimulator of integrins including LFA-1 (50–52) (**Figure 2**). Further we found that the overexpression of RapL “slowed” T-cell motility in D011.10 transgenic T cells in lymph nodes (LNs), an effect reversed by the L224A mutation needed for binding to SKAP1 (44). This implicated RapL and SKAP1 as co-regulators of the “stop-signal’ in T cells. Furthermore, the addition of an N-terminal myr-tag to SKAP1 promoted the constitutive binding of RapL to the cell membrane and replaced the need for TCR ligation in the activation of LFA-1 in T cells. In keeping with this theme, Kliche and coworkers found that the disruption of the ADAP/SKAP1 binding led to a displacement of Rap1 from the plasma membrane and that membrane-targeted SKAP1 induced T-cell adhesion even in the absence of TCR-mediated ligation (58).

In terms of micro-cluster formation, the SKAP1 SH3 domain and associated ADAP have also been reported to stabilize the formation of SLP-76 micro-clusters (59, 60). We also found that LFA1 ligation and the activation of FAK1 to phosphorylate LAT led to the recruitment of SKAP1 to the signalosome (25).

In a minimal model, SKAP1 acts as a chaperone that transports RapL to the PM to interact with the GTP-binding protein Rap1. The RapL–Rap complex (including SKAP1) then binds to the αL chain of LFA-1, promoting its clustering and avidity for its ligand (46, 61). Complementary to this, the β2 chain binds to Kindlins (1, 2, and 3) and TALIN that, in turn, binds directly to Rap1-interacting molecule (RIAM), Paxillin, and Vinculin. TALIN is a high-molecular-weight cytoskeletal protein that links integrins to the actin cytoskeleton (62). Although less well-understood, kindlin proteins are also integrin regulators where their loss or mutations result in defective integrin activation (63). The TALIN unfolded helical bundle R2R3 also binds to RIAM, a site distinct from the vinculin binding site (64). Furthermore, SKAP1 also binds to RIAM (58, 65–67). In this context, it cannot be excluded that SKAP1 acts as a shuttle or chaperone for RIAM. In keeping with these observations, we found that SKAP1 knockout T cells show reduced TALIN-1 and RIAM translocation to the IS (61). Furthermore, non-cleavable TALIN can rescue the defect in the T-cell conjugation of *skap1-/-* T cells (61). Overall, the β2 chain complex promotes the unfolding of LFA-1 to intermediate- and high- affinity forms, while the αL chain-associated SKAP1–RapL–Rap1 complex promotes LFA-1 clustering for high-avidity binding and participates in promoting greater affinity. Further studies will be needed to fully understand the mechanisms underlying these different interactions and their potential cooperative roles in regulating cell adhesion and migration.

## SKAP1 dimerization

One intriguing feature of SKAP1 is its ability to form dimers as mediated by the N-terminal dimerization domain (DM) (43) (**Figure 2**). We showed that both SKAP1 and related SKAP2 [SKAP-R (related) or SKAP-Hom] can form homo- and heterodimers in cells (43, 59, 60). In our hands, homodimer formation of SKAP1 is mediated by residues A17 to L21 in the N-terminal region (43). Intriguingly, both RapL and RIAM have been reported to bind to a similar general region in SKAP1 (44, 65). This begs the question whether dimer formation controls the binding and engagement of the RapL or RIAM pathways in integrin activation. On one level, we found that SKAP1 dimer formation was not needed for RapL binding since dimerization mutants still bind to RapL (43). Others have reported that SKAP1 dimers stabilize the formation and movement of micro-clusters of the key adaptor SLP-76 (59). In this model dimer, they found that dimer formation enabled adhesion *via* the TCR by mechanisms that were independent of RIAM, TALIN, and beta integrin activation. Different mechanisms may therefore be at play that determine the way in which SKAP1 and its dimerization regulate adhesion or other functions. In the context of integrin activation, SKAP1 primarily regulates cluster formation and, to a lesser extent, affinity, while RIAM, KINDLINs, TALIN, and other proteins primarily mediate LFA-1 conformation and affinity changes. It is still unknown whether dimer formation influences PLK1-mediated effects on cell cycling. Overall, these findings highlight the complexity of SKAP1 cellular signaling and the need for further research to fully understand the role of SKAP1 and its dimerization in various cellular functions.

## SKAP1, polo-like kinase, and the cell cycle

In this context, we have found that SKAP1 can also regulate other pathways and cell functions. We found that SKAP1 is phosphorylated by and binds to polo-like kinase (PLK1) for the optimal cycling of T cells (**Figure 2**) (68). PLK1 is a serine/threonine kinase that regulates multiple steps of mitosis and the cell cycle progression of mammalian cells. Among multiple kinases including CDK1, CDK2, MAPK, Aurora B, CAMK, PLK3, PLK1, MST1, and ZAP-70, only PLK1 could phosphorylate SKAP1-GST *in vitro*. Furthermore, PLK1 bound to the N-terminal residue serine 31 (S31) of SKAP1 and the interaction is needed for optimal PLK1 kinase activity in T cells (68). Furthermore, siRNA knock-down of SKAP1 reduced the rate of T-cell division concurrent with a delay in the expression of PLK1, Cyclin A, and pH3. Reconstitution of KD cells with WT SKAP1, but not the SKAP1 S31 mutant, restored normal cell division. SKAP1–PLK1 binding is also seen to be dynamically regulated during the cell cycle of T cells. Our findings identified a novel role for SKAP1 in the regulation of PLK1 and optimal cell cycling needed for T-cell clonal expansion in response to antigenic activation (68).

## SKAP1 and the ERK pathway

Lastly, SKAP1 also influences the activation of the p21^ras^–extracellular signal-regulated kinase (ERK) pathway. p21^ras^ activity is regulated by several mediators that include the guanyl releasing protein 1 (RasGRP1). RasGRP1 exchanges GDP for active GTP on p21^ras^ in a cascade that activates ERKs (69, 70). The Mustelin and Rudd labs independently reported that the C-terminus of SKAP1 binds to RasGRP1 (43, 68). PLK1 and RasGRP1 therefore bind to opposite ends of SKAP1. Furthermore, *skap1-/-* primary T cells had increased RasGRP1 in the trans-Golgi network (TGN) following CD3 ligation where p21^ras^ becomes activated (43). Consistent with this, SKAP1 overexpression impaired Ras-Erk activation with reduced AP-1 transcriptional activity (43). In T cells, the AP-1 transcription factor regulates a wide range of genes related to differentiation and proliferation. However, others have reported that SKAP1 positively regulates the ERK pathway in the T-cell line Jurkat (71). In this pathway, tyrosine 271 played a central role for interaction with both Fyn kinase and adapter protein GRB-2 to mediate mitogen-activated protein kinase activation. In this context, the binding of SKAP1 to GRB2 has also been noted in our lab as mediated by LFA-1 cross-linking and activation of kinases FAK1 and Pyk2 (25). Each kinase phosphorylated a key site at Y-171 on LAT leading to the recruitment of the GRb2-SKAP1 complex. It is possible that the opposing effects of SKAP1 on ERK activation and adhesion are dependent on the concentration of SKAP1 within the cell, and on the presence or absence of other interacting proteins. Further research will be needed to fully understand the complex role of SKAP1 in these pathways.

## SKAP1 and biology

The physiological impact of SKAP1 in regulating immunity is also being studied, although our current understanding is limited due to the early stage of research in this area. Using a mouse model of collagen-induced arthritis (CIA), we showed that *skap1-/-* mice are resistant to the induction of arthritis CIA (69). This was observed in terms of both the incidence of disease and its severity. Furthermore, we noted a marked reduction of joint infiltrating T cells, in particular TH17-like cells. SKAP1 therefore represents a potential target in the therapeutic intervention in autoimmune and inflammatory diseases (69). Lakkis et al. found that skap1 deficiency prolonged allograft survival; it did not seem to alter effector T-cell migration to pancreatic islet allografts (70). Lastly, in certain tumor models, *skap1-/-* mice may also be more resistant to tumor growth (71). Genome-wide association studies (GWAS) have identified SKAP1 as one of eight risk loci for endometrial cancer (72, 73). It is presently unclear how one can reconcile these different findings. It is possible that differences in TCR ligation versus the involvement of chemokines differentially regulates the activity of SKAP1 and its function in different T-cell subsets and in different contexts.

## Conclusion

Overall, while SKAP1 is an immune cell adaptor that regulates T-cell adhesion and optimal cell growth, much remains to be discovered about the physiological impact of SKAP1 in regulating immunity. Further research is needed to fully understand the full range of mechanisms by which SKAP1 regulates T-cell signaling and immune function as well as the downstream effects of SKAP1 activation or inactivation. This may involve assessing the impact of SKAP1 on specific immune cell subsets or its place in various affected disease states such as autoimmunity, transplant rejection, and cancer. Overall, data so far demonstrate that it is an adaptor with multiple functions associated with different regions of the protein. Continued research on SKAP1 will provide important insights into the regulation of immune function and will have implications for the development of new therapies to treat disease states.

## Author contributions

All authors listed have made a substantial, direct, and intellectual contribution to the work, and approved it for publication.

## References

[1] Trowsdale J. The MHC, disease and selection. Immunol Lett (2011) 137:1–8. doi: 10.1016/j.imlet.2011.01.002 21262263

[2] Hennecke J Wiley DC. T Cell receptor MHC interactions up close. Cell (2001) 104:1–4. doi: 10.1016/S0092-8674(01)00185-4 11163234

[3] Hogg N Laschinger M Giles K McDowall A. T-Cell integrins: more than just sticking points. J Cell Sci (2003) 116:4695–705. doi: 10.1242/jcs.00876 14600256

[4] Wilkinson B Downey JS Rudd CE. T-Cell signalling and immune system disorders. Expert Rev Mol Med (2005) 7:1–29. doi: 10.1017/S1462399405010264 16364187

[5] Fooksman DR Vardhana S Vasiliver-Shamis G Liese J Blair DA Waite J. Functional anatomy of T cell activation and synapse formation. Annu Rev Immunol (2010) 28:79–105. doi: 10.1146/annurev-immunol-030409-101308 19968559 PMC2885351

[6] Sherman E Barr V Samelson LE. Super-resolution characterization of TCR-dependent signaling clusters. Immunol Rev (2013) 251:21–35. doi: 10.1111/imr.12010 23278738 PMC3539238

[7] Freiberg BA Kupfer H Maslanik W Delli J Kappler J Zaller DM. Staging and resetting T cell activation in SMACs. Nat Immunol (2002) 3:911–7. doi: 10.1038/ni836 12244310

[8] Wong MM Fish EN. Chemokines: attractive mediators of the immune response. Semin Immunol (2003) 15:5–14. doi: 10.1016/S1044-5323(02)00123-9 12495636

[9] Hosseini BH Louban I Djandji D Wabnitz GH Deeg J Bulbuc N. Immune synapse formation determines interaction forces between T cells and antigen-presenting cells measured by atomic force microscopy. Proc Natl Acad Sci U.S.A. (2009) 106:17852–7. doi: 10.1073/pnas.0905384106 PMC2764924 19822763

[10] Rudd CE. Adaptors and molecular scaffolds in immune cell signaling. Cell (1999) 96:5–8. doi: 10.1016/S0092-8674(00)80953-8 9989491

[11] Rudd CE. How the discovery of the CD4/CD8-p56(lck) complexes changed immunology and immunotherapy. Front Cell Dev Biol (2021) 9:626095. doi: 10.3389/fcell.2021.626095 33791292 PMC8005572

[12] Hunter T Sefton BM. Transforming gene product of rous sarcoma virus phosphorylates tyrosine. Proc Natl Acad Sci U.S.A. (1980) 77:1311–5. doi: 10.1073/pnas.77.3.1311 PMC348484 6246487

[13] Hunter T Cooper JA. Epidermal growth factor induces rapid tyrosine phosphorylation of proteins in A431 human tumor cells. Cell (1981) 24:741–52. doi: 10.1016/0092-8674(81)90100-8 6166387

[14] Rudd CE Trevillyan JM Dasgupta JD Wong LL Schlossman SF. The CD4 receptor is complexed in detergent lysates to a protein-tyrosine kinase (pp58) from human T lymphocytes. Proc Natl Acad Sci U.S.A. (1988) 85:5190–4. doi: 10.1073/pnas.85.14.5190 PMC281714 2455897

[15] Barber EK Dasgupta JD Schlossman SF Trevillyan JM Rudd CE. The CD4 and CD8 antigens are coupled to a protein-tyrosine kinase (p56lck) that phosphorylates the CD3 complex. Proc Natl Acad Sci U.S.A. (1989) 86:3277–81. doi: 10.1073/pnas.86.9.3277 PMC287114 2470098

[16] Rudd CE. CD4, CD8 and the TCR-CD3 complex: a novel class of protein-tyrosine kinase receptor. Immunol Today (1990) 11:400–6. doi: 10.1016/0167-5699(90)90159-7 1964053

[17] Veillette A Bookman MA Horak EM Samelson LE Bolen JB. Signal transduction through the CD4 receptor involves the activation of the internal membrane tyrosine-protein kinase p56lck. Nature (1989) 338:257–9. doi: 10.1038/338257a0 2784195

[18] Wei Q Brzostek J Sankaran S Casas J Hew LS Yap J. Lck bound to coreceptor is less active than free lck. Proc Natl Acad Sci U.S.A. (2020) 117:15809–17. doi: 10.1073/pnas.1913334117 PMC7355011 32571924

[19] Chan AC Kadlecek TA Elder ME Filipovich AH Kuo WL Iwashima M. ZAP-70 deficiency in an autosomal recessive form of severe combined immunodeficiency. Science (1994) 264:1599–601. doi: 10.1126/science.8202713 8202713

[20] Smith-Garvin JE Koretzky GA Jordan MS. T Cell activation. Annu Rev Immunol (2009) 27:591–619. doi: 10.1146/annurev.immunol.021908.132706 19132916 PMC2740335

[21] Kennedy JS Raab M Rudd CE. Signaling scaffolds in immune cells. Cell Calcium (1999) 26:227–35. doi: 10.1054/ceca.1999.0069 10643561

[22] Samelson LE. Signal transduction mediated by the T cell antigen receptor: the role of adapter proteins. Annu Rev Immunol (2002) 20:371–94. doi: 10.1146/annurev.immunol.20.092601.111357 11861607

[23] Raab M da Silva AJ Findell PR Rudd CE. Regulation of vav-SLP-76 binding by ZAP-70 and its relevance to TCR zeta/CD3 induction of interleukin-2. Immunity (1997) 6:155–64. doi: 10.1016/S1074-7613(00)80422-7 9047237

[24] Berg LJ Finkelstein LD Lucas JA Schwartzberg PL. Tec family kinases in T lymphocyte development and function. Annu Rev Immunol (2005) 23:549–600. doi: 10.1146/annurev.immunol.22.012703.104743 15771581

[25] Raab M Lu Y Kohler K Smith X Strebhardt K Rudd CE. LFA-1 activates focal adhesion kinases FAK1/PYK2 to generate LAT-GRB2-SKAP1 complexes that terminate T-cell conjugate formation. Nat Commun (2017) 8:16001. doi: 10.1038/ncomms16001 28699640 PMC5510181

[26] Wang H Rudd CE. SKAP-55, SKAP-55-related and ADAP adaptors modulate integrin-mediated immune-cell adhesion. Trends Cell Biol (2008) 18:486–93. doi: 10.1016/j.tcb.2008.07.005 PMC3512129 18760924

[27] Takagi J Petre BM Walz T Springer TA. Global conformational rearrangements in integrin extracellular domains in outside-in and inside-out signaling. Cell (2002) 110:599–11. doi: 10.1016/S0092-8674(02)00935-2 12230977

[28] Walling BL Kim M. LFA-1 in T cell migration and differentiation. Front Immunol (2018) 9. doi: 10.3389/fimmu.2018.00952 PMC5943560 29774029

[29] Feigelson SW Pasvolsky R Cemerski S Shulman Z Grabovsky V Ilani T. Occupancy of lymphocyte LFA-1 by surface-immobilized ICAM-1 is critical for TCR- but not for chemokine-triggered LFA-1 conversion to an open headpiece high-affinity state. J Immunol (2010) 185:7394–404. doi: 10.4049/jimmunol.1002246 21078912

[30] Wabnitz GH Lohneis P Kirchgessner H Jahraus B Gottwald S Konstandin M. Sustained LFA-1 cluster formation in the immune synapse requires the combined activities of l-plastin and calmodulin. Eur J Immunol (2010) 40:2437–49. doi: 10.1002/eji.201040345 20683899

[31] Fagerholm S Hilden TJ Gahmberg CG. Lck tyrosine kinase is important for activation of the CD11a/CD18-integrins in human T lymphocytes. Eur J Immunol (2002) 32:1670–8. doi: 10.1002/1521-4141(200206)32:6<1670::AID-IMMU1670>3.0.CO;2-M 12115650

[32] da Silva AJ Li Z de Vera C Canto E Findell P Rudd CE. Cloning of a novel T-cell protein FYB that binds FYN and SH2-domain-containing leukocyte protein 76 and modulates interleukin 2 production. Proc Natl Acad Sci U.S.A. (1997) 94:7493–8. doi: 10.1073/pnas.94.14.7493 PMC23849 9207119

[33] Liu J Kang H Raab M da Silva AJ Kraeft SK Rudd CE. FYB (FYN binding protein) serves as a binding partner for lymphoid protein and FYN kinase substrate SKAP55 and a SKAP55-related protein in T cells. Proc Natl Acad Sci U.S.A. (1998) 95:8779–84. doi: 10.1073/pnas.95.15.8779 PMC21153 9671755

[34] Raab M Kang H da Silva A Zhu X Rudd CE. FYN-T-FYB-SLP-76 interactions define a T-cell receptor zeta/CD3-mediated tyrosine phosphorylation pathway that up-regulates interleukin 2 transcription in T-cells. J Biol Chem (1999) 274:21170–9. doi: 10.1074/jbc.274.30.21170 10409671

[35] Veale M Raab M Li Z da Silva AJ Kraeft SK Weremowicz S. Novel isoform of lymphoid adaptor FYN-t-binding protein (FYB-130) interacts with SLP-76 and up-regulates interleukin 2 production. J Biol Chem (1999) 274:28427–35. doi: 10.1074/jbc.274.40.28427 10497204

[36] Musci MA Hendricks-Taylor LR Motto DG Paskind M Kamens J Turck CW. Molecular cloning of SLAP-130, an SLP-76-associated substrate of the T cell antigen receptor-stimulated protein tyrosine kinases. J Biol Chem (1997) 272:11674–7. doi: 10.1074/jbc.272.18.11674 9115214

[37] da Silva AJ Janssen O Rudd CE. T Cell receptor zeta/CD3-p59fyn(T)-associated p120/130 binds to the SH2 domain of p59fyn(T). J Exp Med (1993) 178:2107–13. doi: 10.1084/jem.178.6.2107 PMC2191307 7504057

[38] Boras M Volmering S Bokemeyer A Rossaint J Block H Bardel B. Skap2 is required for beta2 integrin-mediated neutrophil recruitment and functions. J Exp Med (2017) 214:851–74. doi: 10.1084/jem.20160647 PMC5339670 28183734

[39] Swanson KD Tang Y Ceccarelli DF Poy F Sliwa JP Neel BG. The skap-hom dimerization and PH domains comprise a 3′-Phosphoinositide-Gated molecular switch. Mol Cell (2008) 32:564–75. doi: 10.1016/j.molcel.2008.09.022 PMC2628593 19026786

[40] Marie-Cardine A Bruyns E Eckerskorn C Kirchgessner H Meuer SC Schraven B. Molecular cloning of SKAP55, a novel protein that associates with the protein tyrosine kinase p59 fyn in human T-lymphocytes*. J Biol Chem (1997) 272:16077–80. doi: 10.1074/jbc.272.26.16077 9195899

[41] Duke-Cohan JS Kang H Liu H Rudd CE. Regulation and function of SKAP-55 non-canonical motif binding to the SH3c domain of adhesion and degranulation-promoting adaptor protein. J Biol Chem (2006) 281:13743–50. doi: 10.1074/jbc.M508774200 16461356

[42] Kang H Freund C Duke-Cohan JS Musacchio A Wagner G Rudd CE. SH3 domain recognition of a proline-independent tyrosine-based RKxxYxxY motif in immune cell adaptor SKAP55. EMBO J (2000) 19:2889–99. doi: 10.1093/emboj/19.12.2889 PMC203341 10856234

[43] Raab M Strebhardt K Rudd CE. Immune adaptor protein SKAP1 (SKAP-55) forms homodimers as mediated by the n-terminal region. BMC Res Notes (2018) 11:869. doi: 10.1186/s13104-018-3976-3 30522503 PMC6282339

[44] Raab M Wang H Lu Y Smith X Wu Z Strebhardt K. T Cell receptor "inside-out" pathway *via* signaling module SKAP1-RapL regulates T cell motility and interactions in lymph nodes. Immunity (2010) 32:541–56. doi: 10.1016/j.immuni.2010.03.007 PMC3812847 20346707

[45] Raab M Smith X Matthes Y Strebhardt K Rudd CE. SKAP1 PH domain determines RAPL membrane localization and Rap1 complex formation for TCR activation of LFA-1. J Biol Chem (2011) 2011:29663–70286. doi: 10.1074/jbc.M111.222661 PMC3191007 21669874

[46] Wang H Moon EY Azouz A Wu X Smith A Schneider H. SKAP-55 regulates integrin adhesion and formation of T cell-APC conjugates. Nat Immunol (2003) 4:366–74. doi: 10.1038/ni913 12652296

[47] Wang H McCann FE Gordan JD Wu X Raab M Malik TH. ADAP-SLP-76 binding differentially regulates supramolecular activation cluster (SMAC) formation relative to T cell-APC conjugation. J Exp Med (2004) 200:1063–74. doi: 10.1084/jem.20040780 PMC2211848 15477347

[48] Jo EK Wang H Rudd CE. An essential role for SKAP-55 in LFA-1 clustering on T cells that cannot be substituted by SKAP-55R. J Exp Med (2005) 201:1733–9. doi: 10.1084/jem.20042577 PMC2213273 15939789

[49] Wang H Liu H Lu Y Lovatt M Wei B Rudd CE. Functional defects of SKAP-55-deficient T cells identify a regulatory role for the adaptor in LFA-1 adhesion. Mol Cell Biol (2007) 27:6863–75. doi: 10.1128/MCB.00556-07 PMC2099233 17646386

[50] Raab M Smith X Matthess Y Strebhardt K Rudd CE. SKAP1 protein PH domain determines RapL membrane localization and Rap1 protein complex formation for T cell receptor (TCR) activation of LFA-1. J Biol Chem (2011) 286:29663–70. doi: 10.1074/jbc.M111.222661 PMC3191007 21669874

[51] Katagiri K Hattori M Minato N Kinashi T. Rap1 functions as a key regulator of T-cell and antigen-presenting cell interactions and modulates T-cell responses. Mol Cell Biol (2002) 22:1001–15. doi: 10.1128/MCB.22.4.1001-1015.2002 PMC134636 11809793

[52] Shimonaka M Katagiri K Nakayama T Fujita N Tsuruo T Yoshie O. Rap1 translates chemokine signals to integrin activation, cell polarization, and motility across vascular endothelium under flow. J Cell Biol (2003) 161:417–27. doi: 10.1083/jcb.200301133 PMC2172897 12707305

[53] Takagi J Springer TA. Integrin activation and structural rearrangement. Immunol Rev (2002) 186:141–63. doi: 10.1034/j.1600-065X.2002.18613.x 12234369

[54] Kinashi T. Intracellular signalling controlling integrin activation in lymphocytes. Nat Rev Immunol (2005) 5:546–59. doi: 10.1038/nri1646 15965491

[55] Katagiri K Maeda A Shimonaka M Kinashi T. RAPL, a Rap1-binding molecule that mediates Rap1-induced adhesion through spatial regulation of LFA-1. Nat Immunol (2003) 4:741–8. doi: 10.1038/ni950 12845325

[56] Pawson T Nash P. Protein-protein interactions define specificity in signal transduction. Genes Dev (2000) 14:1027–47. doi: 10.1101/gad.14.9.1027 10809663

[57] Yamamoto E Domanski J Naughton FB Best RB Kalli AC Stansfeld PJ. Multiple lipid binding sites determine the affinity of PH domains for phosphoinositide-containing membranes. Sci Adv (2020) 6:eaay5736. doi: 10.1126/sciadv.aay5736 32128410 PMC7030919

[58] Kliche S Breitling D Togni M Pusch R Heuer K Wang X. The ADAP/SKAP55 signaling module regulates T-cell receptor-mediated integrin activation through plasma membrane targeting of Rap1. Mol Cell Biol (2006) 26:7130–44. doi: 10.1128/MCB.00331-06 PMC1592884 16980616

[59] Ophir MJ Liu BC Bunnell SC. The n terminus of SKAP55 enables T cell adhesion to TCR and integrin ligands *via* distinct mechanisms. J Cell Biol (2013) 203:1021–41. doi: 10.1083/jcb.201305088 PMC3871428 24368808

[60] Liu H Purbhoo MA Davis DM Rudd CE. SH2 domain containing leukocyte phosphoprotein of 76-kDa (SLP-76) feedback regulation of ZAP-70 microclustering. Proc Natl Acad Sci U.S.A. (2010) 107:10166–71. doi: 10.1073/pnas.0909112107 PMC2890474 20534575

[61] Lim D Lu Y Rudd CE. Non-cleavable talin rescues defect in the T-cell conjugation of T-cells deficient in the immune adaptor SKAP1. Immunol Lett (2016) 172:40–6. doi: 10.1016/j.imlet.2016.02.004 PMC4860717 26905930

[62] Wegener KL Partridge AW Han J Pickford AR Liddington RC Ginsberg MH. Structural basis of integrin activation by talin. Cell (2007) 128:171–82. doi: 10.1016/j.cell.2006.10.048 17218263

[63] Calderwood DA Campbell ID Critchley DR. Talins and kindlins: partners in integrin-mediated adhesion. Nat Rev Mol Cell Biol (2013) 14:503–17. doi: 10.1038/nrm3624 PMC4116690 23860236

[64] Goult BT Zacharchenko T Bate N Tsang R Hey F Gingras AR. RIAM and vinculin binding to talin are mutually exclusive and regulate adhesion assembly and turnover. J Biol Chem (2013) 288:8238–49. doi: 10.1074/jbc.M112.438119 PMC3605642 23389036

[65] Menasche G Kliche S Chen EJ Stradal TE Schraven B Koretzky G. RIAM links the ADAP/SKAP-55 signaling module to Rap1, facilitating T-cell-receptor-mediated integrin activation. Mol Cell Biol (2007) 27:4070–81. doi: 10.1128/MCB.02011-06 PMC1900018 17403904

[66] Patsoukis N Bardhan K Weaver JD Sari D Torres-Gomez A Li L. The adaptor molecule RIAM integrates signaling events critical for integrin-mediated control of immune function and cancer progression. Sci Signal (2017) 10. doi: 10.1126/scisignal.aam8298 28831022

[67] Kliche S Worbs T Wang X Degen J Patzak I Meineke B. CCR7-mediated LFA-1 functions in T cells are regulated by 2 independent ADAP/SKAP55 modules. Blood (2012) 119:777–85. doi: 10.1182/blood-2011-06-362269 22117043

[68] Raab M Strebhardt K Rudd CE. Immune adaptor SKAP1 acts a scaffold for polo-like kinase 1 (PLK1) for the optimal cell cycling of T-cells. Sci Rep (2019) 9:10462. doi: 10.1038/s41598-019-45627-9 31320682 PMC6639320

[69] Smith X Taylor A Rudd CE. T-Cell immune adaptor SKAP1 regulates the induction of collagen-induced arthritis in mice. Immunol Lett (2016) 176:122–7. doi: 10.1016/j.imlet.2016.04.007 PMC4965781 27181093

[70] Zhang Q Dai H Yatim KM Abou-Daya K Williams AL Oberbarnscheidt MH. CD8+ effector T cell migration to pancreatic islet grafts is dependent on cognate antigen presentation by donor graft cells. J Immunol (2016) 197:1471–6. doi: 10.4049/jimmunol.1600832 PMC5023061 27357151

[71] Li C Li W Xiao J Jiao S Teng F Xue S. ADAP and SKAP55 deficiency suppresses PD-1 expression in CD8+ cytotoxic T lymphocytes for enhanced anti-tumor immunotherapy. EMBO Mol Med (2015) 7:754–69. doi: 10.15252/emmm.201404578 PMC4459816 25851535

[72] O'Mara TA Glubb DM Amant F Annibali D Ashton K Attia J. Identification of nine new susceptibility loci for endometrial cancer. Nat Commun (2018) 9:3166. doi: 10.1038/s41467-018-05427-7 30093612 PMC6085317

[73] Kho PF Wang X Cuellar-Partida G Dork T Goode EL Lambrechts D. Multi-tissue transcriptome-wide association study identifies eight candidate genes and tissue-specific gene expression underlying endometrial cancer susceptibility. Commun Biol (2021) 4:1211. doi: 10.1038/s42003-021-02745-3 34675350 PMC8531339

